# Insight into the mobilome of *Aeromonas* strains

**DOI:** 10.3389/fmicb.2015.00494

**Published:** 2015-05-27

**Authors:** Marta Piotrowska, Magdalena Popowska

**Affiliations:** Department of Applied Microbiology, Institute of Microbiology, Faculty of Biology, University of WarsawWarsaw, Poland

**Keywords:** *Aeromonas*, mobilome, plasmid, transposon, integron, virulence factor, antibiotic resistance gene, horizontal gene transfer

## Abstract

The mobilome is a pool of genes located within mobile genetic elements (MGE), such as plasmids, IS elements, transposons, genomic/pathogenicity islands, and integron-associated gene cassettes. These genes are often referred to as “flexible” and may encode virulence factors, toxic compounds as well as resistance to antibiotics. The phenomenon of MGE transfer between bacteria, known as horizontal gene transfer (HGT), is well documented. The genes present on MGE are subject to continuous processes of evolution and environmental changes, largely induced or significantly accelerated by man. For bacteria, the only chance of survival in an environment contaminated with toxic chemicals, heavy metals and antibiotics is the acquisition of genes providing the ability to survive in such conditions. The process of acquiring and spreading antibiotic resistance genes (ARG) is of particular significance, as it is important for the health of humans and animals. Therefore, it is important to thoroughly study the mobilome of *Aeromonas* spp. that is widely distributed in various environments, causing many diseases in fishes and humans. This review discusses the recently published information on MGE prevalent in *Aeromonas* spp. with special emphasis on plasmids belonging to different incompatibility groups, i.e., IncA/C, IncU, IncQ, IncF, IncI, and ColE-type. The vast majority of plasmids carry a number of different transposons (Tn*3*, Tn*21*, Tn*1213*, Tn*1721*, Tn*4401*), the 1st, 2nd, or 3rd class of integrons, IS elements (e.g., IS*26*, IS*Pa12*, IS*Pa13*, IS*Kpn8*, IS*Kpn6*) and encode determinants such as antibiotic and mercury resistance genes, as well as virulence factors. Although the actual role of *Aeromonas* spp. as a human pathogen remains controversial, species of this genus may pose a serious risk to human health. This is due to the considerable potential of their mobilome, particularly in terms of antibiotic resistance and the possibility of the horizontal transfer of resistance genes.

## Introduction

Bacteria of the genus *Aeromonas* are common in a variety of environments. They have been isolated from water, mammals, fish, invertebrates, birds, insects, soil (Palumbo et al., 1985; Ceylan et al., 2009) as well as from food (Neyts et al., 2000; Kingombe et al., 2004). However, they mainly inhabit all kinds of aquatic environments, such as rivers, lakes, ponds, estuaries of marine waters, drinking water and groundwater as well as wastewater at various stages of purification (Gordon et al., 2008; Reith et al., 2008; Moura et al., 2012). The genus *Aeromona*s is composed of a large number of species (31 species and 12 subspecies Martin-Carnahan and Joseph, 2005; http://www.bacterio.net/aeromonas.html) but only a few of them have been found to be primarily pathogens of fish and warm-blooded animals, including humans. In fish, mainly mesophilic *A. hydrophila*, *A. veronii* bv. *sobria* and psychrophilic strains of *A. salmonicida* are predominantly responsible for fish infections, e.g., furunculosis (Burr et al., 2005; Dallaire-Dufresne et al., 2014), but *A. caviae*, *A. jandaei*, *A. sobria*, *A. bestiarum* have also been reported to cause several known types of diseases as well as unusual infections, e.g., epizootic ulcerative syndrome (Rahman et al., 2002). Mesophilic *A. hydrophila*, *A. caviae*, and *A. veronii* bv. *sobria* strains are important human pathogens, responsible for a variety of infectious complications in both immunocompetent and immunocompromised individuals. They cause various types of infections of the digestive system, such as gastroenteritis (Holmberg and Farmer, 1984; Figueras, 2005; Edberg et al., 2007), respiratory (Bossi-Küpfer et al., 2007) and genitourinary infections (Al-Benwan et al., 2007; Huang et al., 2007), wound infections, infections of skin and soft tissue (Jorge et al., 1998; Vally et al., 2004; Chim and Song, 2007), sepsis (Ko et al., 2000; Lau et al., 2000; Tsai et al., 2006), eye infections (Khan et al., 2007), and meningitis (Seetha et al., 2004).

The mechanisms of the pathogenicity of *Aeromonas* spp. are not yet well understood, this being a concern because of recent reports of antibiotic resistant clinical strains (Ghenghesh et al., 2008; Wu et al., 2015). Moreover, cases of isolation of pathogenic strains from the environment are increasingly frequent, which can pose a serious threat to public health during natural disasters (Lin et al., 2013). Taking into account the pathogenicity potential of *Aeromonas* spp. and differences in antibiotic resistance profiles it seems reasonable to study the genetic background of these phenomena. In our study we have decided to focus on the mobile part of the *Aeromonas* spp. genome, that is the mobilome, since this topic has not yet been comprehensively reviewed. Based on the current knowledge we have focused in this review on plasmids, which carry a number of transposons, integron-associated gene cassettes and IS elements, and encode such determinants as antibiotic and heavy metal resistance, as well as virulence factors.

## Mobile genetic elements and mechanisms of horizontal gene transfer

The mobilome is the total pool of mobile genes in the genome and consists of mobile genetic elements (MGE), such as plasmids, insertion sequences (IS), transposons, integron-associated gene cassettes and bacteriophages. Plasmids are mostly double-stranded and circular independent replicons of extrachromosomal DNA. They cover a variety of sizes from small, often cryptic plasmids to large megaplasmids with many features allowing them to adapt to different environmental conditions (Table 1). Other MGE are transposable elements (TE) such as the insertion sequences (IS) and transposons (Tn) (Table 2). IS are the most simple TE that reach about 0.5–3 kb and very often are flanked by short sequences of inverted repeats (IR). A transposase gene, encoding the transposition of an IS, is usually located between IRs. Transposons have a more complex structure, because in addition to the transposase, they also harbor various genes responsible for specific phenotypes (Oliver et al., 2013). Integrons are non-replicative genetic elements, which are able to capture and incorporate gene cassettes by site-specific recombination. They are composed of three main elements: the *intI* gene, coding for a site-specific recombinase of the integrase family, specific recombination site *attI*, where a gene cassette may be inserted, and the Pc promoter, managing the transcription of the captured gene (Stokes and Hall, 1989).

**Table 1:** **Plasmids of *Aeromonas* spp., their origin and virulence features, and the presence of resistance determinants and mobile elements**.

**Plasmid**	**Size (kb)**	**Inc^a^ group**	**Antibiotic resistance genes**	**Other MGE located within plasmids**	**Conjugative transfer**	**Special features**	**Source of isolation**	**References**
pRA1	144 kb	IncA/C	*sul2*, *tetA*, *tetR*	- Two IS*26* elements - A truncated IS*Vsa3* element	+ (BHR^b^)	- T4SS - *hip*AB toxin-antitoxin gene cluster	Sample type ND (*A. hydrophila*, Japan)	Hedges et al., 1985; Fricke et al., 2009
pR148	165.9 kb	IncA/C	*bla*_OXA-10_, *aadA*1, *sul1*, *tet*A, *tetR*, *catA*2 (MDR^c^)	- Tn*21*: *qacH*, *bla*_OXA-10_, *aadA*1, *sul1*, *intI* (class 1 integron) -Tn*1721*: *tetA, tetR*	–	- T4SS - Mercuric resistance operon (*mer*)	Sample type ND (*A. hydrophila*, tilapia farm, Thailand)	del Castillo et al., 2013
ND^d^	ND	IncA/C	*bla*_CMY-2_, *floR*, *tetA*, *strA-B, aadA*7, *sul2*	- Class 1 integron (*intI*, *aadA*7) - Transposon-like element	+ (BHR)	- Mercuric resistance operon (*mer*)	Fish [*A. salmonicida* subsp. *salmonicida*, atlantic salmon (*Salmo salar*), aquaculture, New Brunswick and Nova Scotia, Canada]	McIntosh et al., 2008
pSN254b	152.2 kb	IncA/C	*floR, sul1, bla*_CMY-2_, *aadA*, *tetA, tetR, sul2*	- Tn*21*: IS*4321*, IS*26*, *intI1*, *aadA, sul1, mer*		-T4SS -Mercuric resistance genes (*merA, merB, merD, merE, merP, merR, merT*)	Fish (*A. salmonicida* subsp. *salmonicida*, brook trout, New Brunswick, Canada)	Vincent et al., 2014
pAsa4	166.7 kb	IncA/C -related	*tetE-tetR*, *sul2*	- Tn*21*: mercury resistance, In2 integron encoding resistance to streptomycin/spectinomycin, quaternary ammonia compounds, sulfonamides (*sul2*) and chloramphenicol	± (genes of conjugative transfer)	- T6SS – three genes (*vgrG*, *icmF*, *hcp*)	Fish (*A. salmonicida* subsp. s*almonicida*, brown trout, Eure river, France)	Reith et al., 2008
RA3	45.9 kb	IncU	*sul1*, *catA*2, *aadA*2 (MDR)	- 10.5 kb class I complex integron (*intI*, *sul1*, *catA*2, *aadA*2, *qacE*Δ1, IS*6100*)	+ (BHR)	–	Fish (*A. hydrophila*, diseased fish Japan)	Sørum et al., 2003; Kulinska et al., 2008
pAR-32	ND	IncU	*sul1*, *catA*2, *aadA*2 (MDR)	- In6-like integron (*intI*, *sul1*, *catA*2, *aadA*2, *qacE*Δ1)	+ (BHR)	- pAr-32 is identical to plasmid RA3	Fish (*A. salmonicida*, diseased biwamasu, trout hatchery, Shiga, Japan)	Sørum et al., 2003; Kulinska et al., 2008
pRAS1	45 kb	IncU	*tetA*, *dfrA16*, *sul1* (MDR)	- Truncated Tn*1721*—5.4 kb *Eco*RI fragment (*tetA*) - In4-like integron (*intI*, *dfrA16, qacEΔ1, sul1*, IS*6100*)	+ (BHR)	–	Fish [*A. salmonicida*, atlantic salmon (*Salmo salar*) with furunculosis, fish farm, Bergen, Norway]	Sandaa and Enger, 1994; Sørum et al., 2003
pASOT, pASOT2, pASOT3	47 kb, 39 kb	IncU	*tetA*	- Tn*1721* – 5.4 kb *Eco*RI fragment (*tetA*)	+ (BHR)	–	Fish (*A. salmonicida*, furunculosis outbreaks, Scotland)	Adams et al., 1998; Rhodes et al., 2000
pFBAOT1-17	large plasmid	IncU – 3,4,5,7,9, 11	*tetA*	- Tn*1721* (*tetA*) in all plasmids	+ (pFBAOT3 to -7, -9, and -11 BHR)	- pFBAOT7 is identical to plasmid pFBAOT11	Environment - untreated hospital effluent, fish farm (*A. hydrophila* and *A. veronii*, United Kingdom)	Rhodes et al., 2000
pFBAOT6	84.7 kb	IncU	*tetA*, *sul1*, *aadA*2 (MDR)	- IS*630* family - In4-like integron (*intI*, *aadA*2, *qacEΔ1*, *sul1*, IS*6100*) - 43-kb Tn*1721*-based transposone - 6.5-kb Tn*3*	+	- First 31 kb (core genes) – Putative replication, maintence and transfer functions - 54 kb – genetic load	Environment - untreated hospital effluent (*A. punctata*, United Kingdom)	Rhodes et al., 2004
pAS37, p42	55 kb, 20 kb	IncU	*qnrS*2	- Mobile insertion cassette (mic) - transposone-like structure - (*qnrS2*)	ND	–	Environment – river (*A. punctata* and *A. media*, Seine River, Paris, France)	Cattoir et al., 2008
p34	80 kb	IncU	*qnrS*2, *bla*_OXA-1_, *aac(6')-Ib-cr, catB3* (MDR)	- Mobile insertion cassette (mic) - transposone-like structure - (*qnrS2*) - IN37-like integron *(intI*, *aac(6')-Ib-cr*, *bla*_OXA-1_, *catB*3)	ND	–	Environment - lake (*A. allosaccharophila*, Lugano lake, Lugano, Switzerland)	Picão et al., 2008
pP2G1	26.6 kb	IncU	*aac(6˘)-Ib-cr, bla_OXA-1_, catB3, mphA-mrx-mphR, qnrS2, arr-3, sul1 (MDR)*	- IS*Kpn9* present upstream of *qnrS2* - In37-like integron (*intI, aac(6¢)-Ib-cr, bla_OXA-1_, catB3* and *arr-3*, *qacEΔ1*, *sul1*) - IS*6100* present upstream of *mphA-mrx-mphR*	–	-Resistance to quaternary ammonium compounds (*qacEΔ1*)	Environment – river (*Aeromonas* sp., Ter River, Ripoll, Spain)	Marti and Balcázar, 2012
ND	20 kb, 35 kb, 40 kb	IncU	*qnrS2*, *aac(6˘)-Ib-cr, sul1*, *dfrA22* (MDR)	- Class 1 integrons (*intI1*): 0.75 kb (*sul1*)	ND	- *qnrS2*, *aac(60)-Ib-cr* (20 kb plasmid) - MDR; - *qnrS2*, *sul1*, *dfrA22* (35 kb plasmid) - *qnrS2* (40 kb plasmid)	Fish (*A. sorbia* and *A. hydrophila*, koi carps - Czech Republic, ornamental fish -Vietnam and Thailand)	Dobiasova et al., 2014
pJA8102-1	11.8 kb	IncQ-2	*tetC*	–	± (Genes of conjugative transfer)	–	Sample type ND (*A. salmonicida*, Japan)	Aoki et al., 1986
pRAS3.1 pRAS3.2	11.8 kb, 12 kb	IncQ-2	*tetC*	–	± (Mobilizable - *mobA*, *mobC*, *mobD*, *mobE*)	- pRAS3.1 and pRAS3.2. putatively variants of the same plasmid pRAS3	Fish [*A. salmonicida* subs. *salmonicida*, atlantic salmon (*Salmo salar*) with furunculosis, fish farm, Norway]	L'Abée-Lund and Sørum, 2002; Loftie-Eaton and Rawlings, 2009
pBRST7.6	7.6 kb	IncQ-3	*qnrS2*	–	± (Mobilizable - *mobB*, *mobC*)	–	Fish (*A. hydrophila*, UDS-affected *Channa punctatus*)	Majumdar et al., 2011
pAHH04	7.2 kb	IncQ	*qnrS2*	–	± (Mobilizable - *mobA*, *mobC*)	–	Fish, environment [*A. hydrophila*, water and diseased Glowlight tetra (*Hemigrammus erythrozonus*), South Korea]	Han et al., 2012c
pAQ2-1, pAQ2-2	6.9 kb	ColE – type	*qnrS2*	- Mobile insertion cassette (mic) element (*qnrS2*)	–	–	Fish [*A. sobria* and *A. hydrophila*, diseased fish Medaka (*Oryzias latipes*) and Congo tetra (*Phenacogrammus interruptus*), South Korea]	Han et al., 2012a
pAHH01	8.9 kb	ColE2 – type	*tetE, tetR*	–	± (Mobilizable - *mob*)	– *relE/B* (toxin-antitoxin)	Fish [*A. hydrophila*, diseased fish cherry salmon (*Oncorhynchus masou masou*), South Korea]	Han et al., 2012b
ND	40 kb	IncFIB	*bla*_CTX-M-15_	- Class 1 (*intI1*) and class 2 (*intI2*) integrons	+	–	Mussel [*A. caviae*, Mediterranean mussel (*Mytilus galloprovincialis*), Adriatic Sea, Kaštela Bay, Croatia]	Maravić et al., 2013
ND	Large plasmids (>30 kb)	ND	*tetA, tetD, tetE, dhfr, catB, aadA1a*	- Class 1 integrons (*intI1*) (in 27 plasmids) - 10 plasmids with *tetA* and *intI* integrons with two or more inserted ARG	+ (- 17 plasmids 110–160 kb)	Plasmid profiles: - A - at least one plasmid >30 kb; - B - one plasmid 2.3–20 kb - C - two plasmids 6.5–15 kb - D - three to nine plasmids 3–25 kb	Fish, environment – water, sediment (*Aeromonas* spp., Denmark)	Schmidt et al., 2001
pTf28	60 kb	ND	*bla*_GES-7_, *aacA4*	- Class 1 integrons (*intI1*, *bla*_GES-7_, *aacA4*)	± (Mobilizable – *mobA*)	–	Environment – water (*A. veronii*, Seine River, Paris, France)	Girlich et al., 2011
pAB5S9	24.7 kb	ND	*floR, sul2, strA-strB, tetR-tetY* (MDR)	- 7.5 kb sequence showed 100% identity to three non-contiguous segments of the SXT element of *Vibrio cholera* (comprised *floR* gene flanked upstream by a complete and downstream by a truncated IS*CR*2 element)	+	–	Environment – river sediment (*A. bestiarum*, freshwater stream, North Brittany, France; New Brunswick and Quebec, Canada)	Gordon et al., 2008; Vincent et al., 2014
pAsa5	155 kb	ND	not detected	–	± (Genes of conjugative transfer)	- T3SS - effector proteins (AopH, AopO, Ati2), chaperones (SycH, SycO, Ati1)	Fish (*A. salmonicida* subsp. *salmonicida*, brown trout, Eure river, France)	Reith et al., 2008; Daher et al., 2011; Dallaire-Dufresne et al., 2013; Vanden Bergh et al., 2013
pAsa6	18.5 kb	ND	not detected	- Eight transposases: six complete and two partial IS sequences	– (Also not mobilizable)	- Two putative truncated genes of sulfatases - *aopH* and *sycH* (T3SS) – virulence factors	Fish [*A. salmonicida* subsp. *salmonicida*, diseased turbot (*Scophthalmus maximus*), Portugal]	Najimi et al., 2009
pAsal1	6.4 kb	ND	–	- IS*AS11*	± (Mobilizable – *mobA*)	- T3SS effector protein (AopP)	Fish (*A. salmonicida* subsp. s*almonicida*, arctic char, Switzerland)	Fehr et al., 2006; Jones et al., 2012; Trudel et al., 2013
pAsal1B	9.0 kb	ND	–	- 2614 bp IS belongs to the IS*21* family inserted in the *mobA* gene	–	- Combination of pAsal1 and IS*AS5*	Sample type ND *(A. salmonicida* subsp. s*almonicida*, France)	Trudel et al., 2013
pASvirA	140 kb	ND	ND	–	ND	- T3SS genes - AexT toxin	Fish [*A. salmonicida*, arctic char (*Savelinus alpines*) with furunculosis]	Stuber et al., 2003
ND	>55 kb	ND	ND	- Class 1 integrons (*intI1*)	+	–	Fish, environment – water, sediment (*Aeromonas* spp., catfish, fish farm, Vietnam)	Nguyen et al., 2014

a *Inc group, Incompatibility group*;

b *BHR, broad host range plasmid*;

c *MDR, multidrug resistance*;

d *ND, not determined; “±”- conjugative transfer not determined or mobilizable*.

**Table 2:** **Other than plasmid borne mobile genetic elements of *Aeromonas* spp**.

**Mobile genetic element**	**Characteristics**	**References**
Tn*3*	- Tn*3*-*tnpR*/IS*Kpn8*/*bla*_KPC-2_/IS*Kpn6*	Picão et al., 2013
Tn*21*	- *qacH, bla_OXA-10_, aadA1, sul1, intI* - IS*4321*, IS*26*, *intI*, *aadA, sul1, mer* - *mer*	Reith et al., 2008; del Castillo et al., 2013; Vincent et al., 2014
Tn*1721*	*- tetA, tetR*	Rhodes et al., 2000; Sørum et al., 2003; del Castillo et al., 2013
Tn*4401*	*- bla*_KPC-2_	Picão et al., 2013
IS*26*	- Sulfonamide resistance gene - *bla*_PER-1_ (between IS*Pa12* and IS*Pa13* which composed part of Tn*1213*)	Fricke et al., 2009; Girlich et al., 2011
IS*6100*	*mphA-mrx-mphR*	Marti and Balcázar, 2012
ISV*sa3*	- *sul2*	Fricke et al., 2009
IS*Kpn9*	- *qnrS2*	Marti and Balcázar, 2012
Class I integrons	- 10.5 kb class I complex integron (*intI, sul1, catA2, aadA2, qacEΔ1*, IS*6100*) - 0.75 kb integron (*sul1*) - *bla*_GES-7_, *aacA4* - *aadA*7 - *bla*_VEB-1_ - *bla*_SHV-12_	Kulinska et al., 2008; McIntosh et al., 2008; Girlich et al., 2011; Dobiasova et al., 2014
IN2-like integron (class I)	- Resistance to streptomycin/spectinomycin, quaternary ammonia compounds, sulfonamides (*sul2*) and chloramphenicol	Reith et al., 2008
IN4-like (class I)	- *intI, dfrA16, qacEΔ1, sul1*, IS*6100* - *intI, aadA2, qacEΔ1, sul1*, IS*6100*	Sørum et al., 2003; Rhodes et al., 2004
IN6-like (class I)	*intI, sul1, catA2, aadA2, qacEΔ1*	Kulinska et al., 2008
IN37-like (class I)	- *intI*, *aac(6')-Ib-cr*, *bla*_OXA-1_, *catB*3 - *intI, aac(6¢)-Ib-cr, bla_OXA-1_, catB3 and arr-3*, *qacEΔ1*, *sul1*	Picão et al., 2008; Marti and Balcázar, 2012
SXT	- *floR* gene flanked upstream by a complete and downstream by a truncated IS*CR2* element	Gordon et al., 2008
Mobile insertion cassette (mic)	- *qnrS2*	Cattoir et al., 2008; Picão et al., 2008; Han et al., 2012a

MGE are ubiquitous among all prokaryotes and play a significant role in horizontal gene transfer (HGT) and interspecies dissemination of resistance and virulence determinants (Brouwer et al., 2011; Oliveira et al., 2014). HGT occurs mainly by three mechanisms: DNA transformation, conjugative transfer involving plasmids, and other conjugative elements (conjugative transposons) and transduction by phages (Thomas and Nielsen, 2005). In the case of *Aeromonas* spp. until now gene transfer by transduction has never been observed. However, *Aeromonas* spp. phages have been identified in various environments, such as *A. hydrophila* phages Aeh1 and Aeh2 from sewage (Chow and Rouf, 1983), *A. hydrophila* phage CC2 from sewage in China (Shen et al., 2012), *A. salmonicida* phage phiAS4 from river in Korea (Kim et al., 2012a), and *A. salmonicida* phage PAS-1 from aquaculture in Korea (Kim et al., 2012b). Additionally, prophages have been also detected in *Aeromonas* spp.: Φ O18P (*Myoviridae*) in *A. media* isolated from a pond in Germany (Beilstein and Dreiseikelmann, 2008), AH1, AH2, AH3, AH4, and AH5 in *A. hydrophila* isolated from epidemic outbreak of catfish in the USA (Hossain et al., 2013). Hossain et al. (2013) detected five putative prophages (AH1-5) located in epidemic-associated regions in the genome. Unfortunately, their studies have not shown the ability of the prophages to transduce any bacterial genes. The other two mechanisms of HGT (DNA transformation and conjugative transfer by plasmids) are ubiquitous among *Aeromonas* spp. and will be the subject of this review. Various MGE, such as plasmids, transposons, or insertion sequences have been isolated from aeromonads, and many of them, regardless of the strain origin, have been found to carry resistance or virulence determinants (Sørum et al., 2003; Dallaire-Dufresne et al., 2014).

### Antibiotic resistance genes on MGE

Over the years, there has been very little research on the *in vitro* susceptibility of *Aeromonas* bacteria isolated from clinical material to various chemotherapeutic agents. Most of the available information is focused on antibiotics used to treat infections caused by *A. hydrophila*, *A. caviae*, and *A. veronii* bv. *sobria*, and it is not yet certain whether these results can be extrapolated to other species of this genus (Fricke et al., 2009; Girlich et al., 2011; Maravić et al., 2013). The Clinical and Laboratory Standards Institute (CLSI) has recently published guidelines for the assessment of the sensitivity of clinical isolates of *Aeromonas* spp. using disk diffusion and MIC tests, but these data are based on testing of the three above- mentioned-, most clinically relevant species of *Aeromonas* (Jorgensen and Hindler, 2007) plus *A. jandaei* and *A. schubertii* (Clinical and Laboratory Standards Institute, 2006). The most commonly administered antibiotics In the treatment of *Aeromonas* infections are ciprofloxacin, levofloxacin, sulfamethoxazole/trimethoprim, amikacin, gentamicin, ciprofloxacin, and trimethoprim (Jones and Wilcox, 1995). Sensitivity analysis of clinical strains demonstrated that more than half of the strains tested were resistant to antibiotics of the following groups: antifolates (sulfamethoxazole), cephalosporins, penicillins (amoxicillin, ampicillin, ampicillin-sulbactam, oxacillin, penicillin, ticarcillin. The susceptibilities were determined by agar dilution or disc diffusion method, respectively, according to CLSI guidelines, but the ARG were not pin-pointed (Lamy et al., 2009; Aravena-Román et al., 2012). However, the susceptibility profile of individual strains can also vary depending on the particular species, different geographical localization and environment in which they occur (Ghenghesh et al., 2008). Such differences could be related to the recommended approach for the treatment of *Aeromonas* infections in different countries.

On the basis of fairly abundant literature data concerning the antibiotic resistance of environmental *Aeromonas* strains, it can be concluded that this phenomenon mostly concerns strains isolated from various water environments, including wastewater (Figueira et al., 2011), natural waters such as rivers (Girlich et al., 2011), lakes (Picão et al., 2008), and estuaries (Fiorentini et al., 1998; Henriques et al., 2006), aquacultures (Schmidt et al., 2001; Jacobs and Chenia, 2007; Yi et al., 2014a,b), and urban drinking water (Carvalho et al., 2012). ARG recently found in water strains encoded resistance to four major groups of antibiotics: β-lactams, quinolones, aminoglycosides, tetracyclines and less frequently to sulfonamides and trimethoprim, chloramphenicol, florfenicol, macrolides, streptogramins, streptothricin, and ansamycins. In general, the resistance profile and the presence of specific resistance genes depends on the particular aquatic environment (Piotrowska and Popowska, 2014). Given the risk to human health, the incidence of ARG is alarming, particularly among *A. hydrophila*, *A. caviae*, and *A. sorbia*, which are considered opportunistic pathogens responsible for infections in both fish and humans (Alcaide et al., 2010; Ottaviani et al., 2011; Shak et al., 2011; Dias et al., 2012; Yi et al., 2014b). The localization of ARG and virulence determinants of *Aeromona*s spp. on MGE such as plasmids, insertion sequences, transposons and mobile integron gene cassettes have been determined by many environmental studies. There is some literature data on the localization of ARG among clinical *Aeromonas* strains. However, in the recent publications, the presence of ARG on plasmids (e.g., MOX, TEM, PSE, and CTX-M β-lactamase genes, *sul1* and *sul2*) has been confirmed, but no characteristics have been provided (Ye et al., 2010; Puah et al., 2013). Moreover, among clinical strains three cases of β-lactamases genes located within integrons: *bla*_VIM_ from *A. caviae* (Adler et al., 2014), *bla*_VIM−4_ from *A. hydrophila* (Libisch et al., 2008) *bla*_IMP_ which also was located on 35-kb plasmid from *A. caviae* have been identified (Neuwirth et al., 2007). Also (Wu et al., 2011) identified the ESBL gene *bla*_PER−3_ in two *A. caviae* isolates. The gene was located in both chromosomes and plasmids. Additionally, there is only one clinical report of gene *qnrS2* that has been found on a plasmid isolated from *A. veronii* (Sánchez-Céspedes et al., 2008). However, all these reports are still sufficient enough to look for any gene-MGE correlation and consequently research on a larger scale should be conducted.

#### Plasmids

Analysis of a number of studies on *Aeromonas* spp. showed the prevalence of different incompatibility groups of plasmids, i.e., IncA/C, IncU, IncQ, IncF, IncI, and ColE-type, with the greatest frequency of the first two groups (Table 1). The vast majority of plasmids carry a number of different determinants such as antibiotic and metal resistance genes or virulence factors. *Aeromonas* spp. very often carry resistance plasmids (R-plasmids) of various length belonging to different incompatibility groups and of worldwide spread. Furthermore, numerous R-plasmids contain multidrug-resistance (MDR) to three or more antimicrobial classes (according to the European Centre of Disease Prevention and Control, Magiorakos et al., 2012). Of particular concern is the fact that most of the isolated plasmids are broad-host-range (BHR), capable of conjugative transfer (*tra* genes) or capable of mobilization (*mob* genes).

Plasmids of the IncA/C incompatibility group have been described as conjugative and BHR, and capable of spreading multidrug-resistance. A variety of isolates among different bacterial genera, such as *Escherichia*, *Salmonella*, *Vibrio*, or *Yersinia* (Winokur et al., 2001; Pan et al., 2008; Guo et al., 2014) of environmental, animal and human origin have been reported to carry these plasmids, which increases public health concerns worldwide (Mataseje et al., 2010). The first member of the IncA/C family was the pRA1 plasmid isolated in 1971 from *A. hydrophila* derived from Japan (Hedges et al., 1985). The complete DNA sequence of this large plasmid (144 kb) revealed that pRA1 and other members of the IncA/C family shared 100 kb of a highly conserved plasmidic backbone with more than 80% nucleotide sequence identity. Among the most important core genes are those that encode type IV secretion-like conjugative transfer operons. A complete set of the type IV secretion system operons was also found on pR148 (MDR plasmid) of IncA/C group isolated from diseased fish from Thailand (del Castillo et al., 2013). Another interesting feature was the *hipAB* toxin-antitoxin gene cluster, which was also partially (*hipAB*-related gene cluster) described in the IncA/C-related plasmid pAsa4, isolated from the fish pathogen *A. salmonicida* subsp. *salmonicida* (Fricke et al., 2009). According to the BLAST database, integrating conjugative elements (ICE) were identified as the closest relatives of IncA/C plasmids (Fricke et al., 2009). Antimicrobial resistance profile of pRA1 is reduced compared with all other IncA/C plasmids sequenced of *Aeromonas* spp., as it is limited to tetracyclines (*tetRA* cluster) and sulfonamides (*sul2*) (McIntosh et al., 2008; del Castillo et al., 2013). The *sul2* gene was located next to the truncated IS*Vsa3* element, which has been observed previously in an IncA/C plasmid isolated from a Spanish *S. enterica* strain (García et al., 2011). Class D *tetRA* gene cluster was found within two IS*26* elements that played a key role in the distribution of ARG on different plasmids (Cullik et al., 2010). In addition to antibiotics, heavy metals are also implicated as potential substances that can co-select antibiotic resistance in the environment, resulting in a frequent presence of heavy metal resistance genes on the same MGE as ARG (Lazar et al., 2002). Mercury resistance operons (*mer*) have been found on IncA/C plasmids such as pR148 or pAsa4 in *Aeromonas* spp. The plasmid isolated from *A. salmonicida* subsp. *salmonicida* showed 100% nucleotide sequence homology to the *mer* operon carried by *S. enterica* IncA/C plasmid pSN254 (McIntosh et al., 2008; del Castillo et al., 2013). The pR148 plasmid also carries genes encoding resistance to quaternary ammonium compounds. Moreover, phylogenetic comparative studies revealed that pR148 is the most closely related to human pathogenic *E. coli* and *Acinetobacter baumanii*. This similarity indicates that the IncA/C group of plasmids was transferred between different genera (McIntosh et al., 2008; Moura et al., 2012). Furthermore, the IncA/C MDR plasmid isolated by McIntosh et al., 2008 carried *floR*, *tetA*, *sul2*, and *strA*/*strB* sequences on a cassette that had 99.9% nucleotide sequence homology to that of the pSN254 plasmid isolated from *S. enterica*. Recently Vincent et al. (2014) identified a 152-kb pSN254b plasmid which is a different variant of pSN254. This MDR plasmid provides resistance to chloramphenicol (*floR*), florfenicol (*floR*), streptomycin (*aadA*), spectinomycin (*aadA*), tetracycline (*tet*), sulfonamide (*sul1*), beta-lactam antibiotics (*bla*_CMY−2_), quaternary ammonium compounds (*sugE2*), and mercury (*merA*, *merB*, *merD*, *merE*, *merP*, *merR*, *merT*). There is no strong correlation between IncA/C plasmids and other MGE, but suggestive associations have been observed and will be described in the next chapter.

Plasmids of the IncU group have been isolated from many clinical and environmental strains of *Esherichia coli* and *Aeromonas* spp. Conjugative and BHR plasmids are members of this group and are also involved in the dissemination of antibiotic resistance among *Aeromona*s spp. This group of plasmids is widely distributed around the world (Table 1) and it has been postulated that they share a conserved backbone structure with a variable region limited to resistance-determining genes (Rhodes et al., 2000; Sørum et al., 2003). The RA3 plasmid (45.9 kb) was isolated from *A. hydrophila* in Japan and serves as the reference plasmid of the IncU group (Kulinska et al., 2008). Functional analysis demonstrated that RA3, as a BHR plasmid, could self-transfer, replicate and be stably maintained in Alpha-, Beta-, and Gammaproteobacteria. RA3, similarly to other members of the IncU group, is a MDR plasmid and contains a 10.5-kb antibiotic resistance region that comprises class I integron with *sul1*, *catA2*, *aadA2*, *qacE* resistance genes. The pAr-32 plasmid, isolated from *A. salmonicida* in Japan in 1970 is very similar to the RA3 plasmid and carries the same integron cassette, which is highly similar to the In6 integron of the pSa plasmid (Sørum et al., 2003). Another IncU plasmid (pRAS1) was isolated from *A. salmonicida* from Norway in 1989 and had the same backbone structure as pAR-32. The region controlling drug resistance in pRAS1 contains two main elements: the complete class 1 In4-like integron with *dfrA16*, *qacE*, *sul1* gene cassette and a fragment of the Tn*1721* transposon carrying *tetA* resistance gene. The study of Schmidt et al. (2001) demonstrated a positive correlation between oxytetracycline resistant strains of *Aeromonas* spp. containing large plasmids, and the presence of *tetA* genes. Among IncU R-plasmids, such as pRAS1, pASOT, or pFBOAT, tetracycline resistance determinants were observed in the complete or truncated Tn*1721* (Adams et al., 1998; Rhodes et al., 2000). In all cases the TetA determinant was located within a 5.5-kb *Eco*RI restriction fragment. Based upon RFLP assessment, antibiotic resistance, and frequency of transfer all these tetracycline resistance encoding plasmids are considered to be closely related to plasmid pIE420 isolated from a German hospital strain of *E. coli* (Rhodes et al., 2000). The study of Rhodes et al. (2004) showed that plasmids pRAS1 and pIE420 are probably identical. These results support the hypothesis that IncU is an evolutionarily narrow group. However, Rhodes et al. (2004) also characterized plasmid pFBOAT6 (84.7 kb), which had a 31-kb region of core genes and a 54-kb region of genetic load, which made this plasmid almost twice as large as the other IncU plasmids. This was due to the presence of a 43-kb resistance region flanked by Tn*1721*. This region is highly similar to those of the pXF51, pIPO2, and pSB102 plasmids found in a plant-associated bacterial hosts. Nevertheless, only several nucleotide differences in the core genes were found between RA3 and pFBAOT6 plasmids.

Many IncU plasmids also harbor quinolone resistance determinants–*qnrS2* and *aac(6′)-Ib-cr* (Table 1). Plasmid-mediated *qnr* genes have been identified in many *Enterobacteriaceae* isolates (Nordmann and Poirel, 2005; Kehrenberg et al., 2006; Pasom et al., 2013) and recently also in *Pseudomonas* spp. (Cayci et al., 2014). In addition, the *qnrS2* gene has been recently detected in *A. caviae* clinical strain isolated from a stool sample collected from a patient with gastroenteritis (Arias et al., 2010). Among the environmental isolates of *Aeromonas* spp., the *qnrS2* gene was found in the following plasmids: pAS37 and p42 from French strains of *A. punctata* and *A. media* (Cattoir et al., 2008), p34 from a Swiss strain of *A. allasacharophila* (Picão et al., 2008), pP2G1 from a Spanish strain (Marti and Balcázar, 2012) and recently isolated unnamed plasmids from Thai strains of *A. sorbia* and *A. hydrophila* (Dobiasova et al., 2014). All of the described plasmids are medium to large in size (20–80 kb) and have an interesting genetic descent. The first two plasmids, pAS37 and p34, contain the *qnrS2* gene as a part of a novel transposon-like genetic structure called the mobile insertion cassette (mic) instead of the transposase gene. Moreover, this specific mobile element has been previously found in a *Bacillus cereus* strain, which makes mic a possible vector of ARG between environmental and clinical pathogens (de Palmenaer et al., 2004). Furthermore, the study of Han et al. (2012a) revealed two small (6.9 kb) plasmids (pAQ2-1 and pAQ2-2) carrying the mic-*qnr2S* structure. These ColE-type plasmids were 99% identical and genes for plasmid replication were organized in a similar way to ColE2-type cryptic plasmids pAsa1, pAsa2, and pAsa3, isolated from *A. salmonicida* subsp. *salmonicida* (Boyd et al., 2003). This observation suggests that these mic-type structures are potential vehicles of plasmid-mediated quinolone resistance determinants among different groups of plasmids in various geographical locations, and more importantly in clinically relevant strains.

Plasmids of the IncQ group are also strongly associated with quinolone resistance that have been identified among *Aeromonas* spp. These small, mobilizable plasmids (5.1–14.2-kb) are BHR and are found in many bacterial species worldwide (Loftie-Eaton and Rawlings, 2009). Among *Aeromonas* spp., two plasmids (pBRST7.6 and pAHH04) isolated from *A. hydrophila* strains from diseased fish and water samples harbored *qnrS2* genes (Majumdar et al., 2011; Han et al., 2012c). In addition, the exogenous pGNB2 plasmid obtained from the wastewater treatment plant in Germany also harbored the *qnrS2* gene (Bönemann et al., 2006). In contrast to IncU plasmid-mediated *qnr* genes, quinolone determinants of the IncQ plasmids were not associated with any mic or integron, and did not harbor any additional resistance determinants. Moreover, the pJA8102-1 plasmid found in *A. salmonicida* from Japan, and pRAS3.1 and pRAS3.2 of Norwich *A. salmonicida* strains carried *tetAR*(C) genes (L'Abée-Lund and Sørum, 2002). However, it is worth to emphasize that pRAS3.1 and pRAS3.2 are considered variants of the same plasmid pRAS3, which was also identified in a Scottish *A. salmonicida* strain and appears to be identical to the R-plasmid pJA8102-2.

In addition to the frequently occurring BHR plasmids that were discussed earlier (IncA/C, IncU, IncQ), plasmids belonging to IncFrepB, IncFIB, IncFIC and IncI groups were also observed in the genus *Aeromona*s (Han et al., 2012b; Moura et al., 2012; Maravić et al., 2013). The study of Maravić et al. (2013) found 40-kb conjugative plasmids described as narrow host range IncFIB group in 11 *A. caviae* strains isolated from Croatian mussels. All the vectors carried the *bla*_CTX−M−15_ gene, encoding ESBL β-lactamase. The same β-lactamase was also identified in IncFIB plasmids isolated from *E. coli*, and a large (210 kb), non-conjugative IncFIA plasmid identified in an *A. hydrophila* strain (Dolejska et al., 2011; Gómez-Garcés et al., 2011).

#### Insertion sequences and transposons

*Aeromonas* spp. often contain transposons located on plasmids and chromosomes (Tn*3*, Tn*21*, Tn*1213*, Tn*1721*, Tn*4401*) as well as insertion sequences (e.g., IS*26*, IS*Pa12*, IS*Pa13*, IS*Kpn8*, IS*Kpn6*) (Tables 1, 2). This makes the mobilome an even more complex structure that likely plays an important role in the dissemination of various resistance and virulence determinants.

McIntosh et al. (2008) found a transposon-like element that contained the *bla*_CMY−2_ β-lactamase gene. This element is known to be widely distributed among foodborne and clinical *Salmonella* strains as well as other *Enterobacteriaceae* in Asia and the United States. Other transposons (e.g., Tn*21*) are involved in the dissemination of ARG and mercury resistance genes (as in pAsa4) between gram-negative bacteria (Liebert et al., 1999). Transposon Tn*21* located on the pR148 plasmid carried *qacH*, *bla*_OXA−10_, *aadA1*, and *sul1* cassette, which showed 100% similarity (when the last gene is excluded) to *Acinetobacter baumanii* AYE genome. A Tn*1721*-like transposon, conferring tetracycline resistance via *tetA/R* genes, was identified on the same plasmid. Tn*1721* belongs to the Tn*501* subfamily and the Tn*3* family of transposons. The involvement of Tn*1721* and Tn*1721*-like elements in the dissemination of the *tetA* gene has been observed in many other studies (Rhodes et al., 2000; Pasquali et al., 2005; Girlich et al., 2010). This transposon is also ubiquitous among IncU plasmids, which form another important group within *Aeromonas* spp.

Furthermore, many transposons and various insertion sequences were identified among *Aeromonas* spp. in association with β-lactamases. The study of Girlich et al. (2011) revealed many ESBL β-lactamases of different genetic backgrounds. The *bla*_SHV−12_ gene was preceded by IS*26* while the *bla*_PER−1_ gene was located between IS*Pa12* and IS*Pa13*, thus forming a part of a composite transposon Tn*1213*. It is also alarming that *bla*_KPC−2_ genes encoding carbapenemases have been isolated from *Aeromonas* spp. recovered from hospital sewage (Picão et al., 2013). The *bla*_KPC−2_ gene was found on the Tn*4401* transposon and Tn*3*-*tnpR*/IS*Kpn8*/*bla*_KPC−2_/IS*Kpn6* array.

#### Integrons

Integrons are widely distributed bacterial genetic elements that are able to acquire gene cassettes frequently containing ARG. Most of them belong to the 1st, 2nd, or 3rd class of integrons and contain *intI1*, *intI2*, or *intI3* integrase genes, respectively (Hall et al., 1999). Integrons harbored by plasmids, transposons and other mobile structures are called “mobile integrons” (MI), because MGE promote their dissemination. For this reason, MI are also involved in spreading antibiotic resistance in the environment (Laroche et al., 2009). Integrons found in *Aeromonas* spp. mainly belong to the class 1 and carry a number of antibiotic resistance gene cassettes (Tables 1, 2). Schmidt et al. (2001) demonstrated that class 1 integrons frequently occurred on oxytetracycline resistance plasmids (most often *tetA*), but they did not observe any strong correlations between integrons and tet genes or any other group of plasmids. Similar results were reported by Jacobs and Chenia (2007), who observed class 1 integrons and tet genes in 68.4% isolates that also harbored different types of plasmid profiles. However, the study of Moura et al. (2012) demonstrated a positive correlation between integrons and FrepB and I1 plasmids isolated from *Aeromonas* spp. from wastewater. Furthermore, Schmidt et al. (2001) reported a close association of sulfadiazine/trimethoprim resistance and class 1 integrons, which manifested in the presence of *sul1* and *dfr* gene cassette inserts in class 1 integrons. Henriques et al. (2006), detected *intI* genes in 21% of *Aeromonas* and 29.6% of *Enterobacteriaceae* isolates. The most often found resistance gene cassettes contained various *aadA* genes, which were also observed in later studies (Moura et al., 2007; Koczura et al., 2014; Sarria-Guzmán et al., 2014). Integrons are also correlated with β-lactamase genes, e.g., genes *bla*_VEB−1_ and *bla*_SHV−12_were located in class 1 integrons on plasmids of different sizes (30–170 kb) (Jacobs and Chenia, 2007; Carvalho et al., 2012). Integrons of class 2 were found in a couple of studies that indicated putative chromosomal location of these integrons (Carvalho et al., 2012). Other integrons belonging to class 1, such as IN2-like, IN4-like, IN6-like, IN37-like, or SXT, as well as many undescribed integrons, have been also reported on *Aeromonas* spp. plasmids (Table 1).

### Virulence factors on MGE

The role of potential virulence factors in the pathogenesis of *Aeromonas* spp. is not yet fully understood. However, several factors probably play important roles in the host infection process. In addition to adhesive factors, the capsular polysaccharide of *Aeromonas* (Khan et al., 2008), bacterial flagella and pili are needed for the first stage of infection. Lysis proteases, which are involved in the second step (metalloproteases, serine proteases, and aminopeptidases) are capable of degrading complex proteins present in the serum and connective tissue (Merino et al., 1996; Han et al., 2008; Imamura et al., 2008; Ottaviani et al., 2011; Puthucheary et al., 2012; John and Hatha, 2013). Many other factors are also likely to play important roles in infections, including specific proteins required for adaptive acid tolerance, biofilm formation and synthesis of autoinducers (e.g., acyl-homoserine lactone) in the quorum sensing process (Jangid et al., 2007) and type three secretion systems (Vanden Bergh et al., 2013). S-layers are also important, as they have the ability to bind different proteins, such as the extracellular matrix proteins fibronectin, laminin, and vitronectin, which provide a defense against the components of the serum and protease digestion (Noonan and Trust, 1997). The main pathogenic factors of *Aeromonas* spp. have also been observed on several plasmids (Table 1). Genes coding for the AexT toxin and three types of secretion systems—type III (T3SS), type IV (T4SS), and type VI (T6SS) have been identified on virulence plasmids (Table 1).

Six virulence plasmids were isolated from strains of the fish pathogen *A. salmonicida* i.e., pASvirA from diseased fish, pAsa4 and pAsa5 from the same French strain, pAsa6 from diseased fish in Portugal (Stuber et al., 2003; Reith et al., 2008; Najimi et al., 2009) and pAsal1 and pAsal1B (Fehr et al., 2006; Jones et al., 2012; Trudel et al., 2013). Three plasmids were large (140–166.7 kb), i.e., pASvirA, pAsa4 and pAsa5, and in addition the latter two harbored conjugative transfer genes. Plasmid pAsa5 contained most of the T3SS genes that have been shown to be required for virulence in *A. salmonicida*. Three putative effector proteins (AopH, AopO, Ati2) and their associated chaperones (SycH, SycO, Ati1) were identified in it. The recommended temperature for growth of *A. salmonicida* by Bergey's Manual of Systematic Bacteriology is 22–28°C. However, several studies demonstrated that culturing at 25°C resulted in a lack of virulence due to the loss of virulence factors, such as the A-layer (Ishiguro et al., 1981), T3SS region (Stuber et al., 2003), or AexT toxine (Najimi et al., 2009). The mechanism of this phenomenon is not fully understood, but Stuber et al. (2003) explained it as a result of the loss of a virulence plasmid. As an example, the loss of plasmid pASvirA is accompanied by the inability of *A. salmonicida* to secrete AexT and loss of virulence toward RTG-2 cells. However, Tanaka et al. (2013) proposed a mechanism of the loss of virulence mediated by IS, wherein rearrangements are caused by recombination of three IS from thermolabile plasmids, e.g., pAsa5 or pASvirA. A consequence of the recombination of IS*AS1*, IS*AS2*, and IS*AS11* is the deletion of the T3SS region in *A. salmonicida*, resulting in the loss of virulence. This is consistent with a previous study that showed a large number of IS in *A. salmonicida* genome being involved in virulence gene disruption with the formation of pseudogenes (Reith et al., 2008). Sequencing and analysis studies of the total genome of *A. salmonicida* subsp. *salmonicida* A449 revealed the occurrence of 88 complete IS sequences of different types: IS*As1*, IS*As2*, IS*As3*, IS*As4*, IS*As5*, IS*As6*, IS*As7*, IS*As8*, IS*As9*, IS*As10*, ISAs11 and a significant number of pseudogenes (170). Many putative transposons and IS sequences, as well as AopH and SycH proteins are also present on pAsa6. This vector is a non-mobilizable 18-kb plasmid with characteristic strong homology to many pAsa5 genes (Najimi et al., 2009). Comparative analyses suggested that pAsa6 might be derived from pAsa5 through a deletion of numerous genes, or conversely, pAsa5 might have been formed as a fusion of a pAsa6-like plasmid with another megaplasmid. This is even more interesting when one considers the fact that both plasmids were isolated in different countries. Three genes of T6SS (*vgrG, icmF, hcp*) were found on pAsa4, but the majority of them were located on the chromosome of *A. salmonicida* subs. *salmonicida* or *A. hydrophila* (Reith et al., 2008). It has been demonstrated that T6SS plays an important role in the pathogenesis of *Aeromonas* strains, in the translocation of hemolysin protein (Hcp) into the host cells (Suarez et al., 2008). In addition, two small mobilizable plasmids, pAsal1 and pAsal1B (6.7 and 9.0 kb, respectively) encoding T3SS effector protein AopP, were recently discovered (Trudel et al., 2013). AopP has been reported to have a inhibitory activity against the NF-κB pathway in cultured cells (Fehr et al., 2006) and has potent pro-apoptotic activity when expressed in cultured mammalian macrophage or epithelial cells (Jones et al., 2012). Trudel et al. (2013) showed that pAsalB is a combination of pAsal1 and ISAS5, where this 2614 bp IS belongs to the IS*21* family inserted in the *mobA* gene sequence of the pAsal1 plasmid.

## Conclusions

Bacteria from the genus *Aeromonas* have a complex mobilome consisting of many different MGE. This review presents the characteristics of more than 26 plasmids belonging to different incompatibility groups, all of which were isolated from environmental strains. Resistance genes have been detected in 21 of them, and 7 meet the MDR criteria for the isolated *Aeromonas* strains. Of particular note are the conjugative broad-host-range plasmids, belonging to the incompatibility group IncA/C, IncU, IncQ, IncF, IncI, and ColE-type. These plasmids are primarily responsible for multi-drug resistance among bacteria both in clinical and natural environments. They harbor resistance genes against antibiotics of key importance in clinical therapy, such as the quinolones, β-lactams, aminoglycosides, tetracyclines and sulfonamide. *Aeromonas* strains causing infections in humans may transfer MGEs carrying resistance genes to pathogenic or opportunistic bacteria in the human microbiome, and thus pose a threat to public health. This *in vivo* transfer has been reported from two clinical outbreaks in France where a 180-kb plasmid carrying the *bla*_TEM−24_ gene has been isolated from *Enterobacter aerogenes* and two *Aeromonas* species: *A. hydrophila* and *A. caviae* (Marchandin et al., 2003; Fosse et al., 2004). The same plasmid has been previously characterized in the case of *Klebsiella pneumonia* and *Pseudomonas aeruginosa* (Giraud-Morin and Fosse, 2003; Marchandin et al., 2000) indicative of their broad host range potential among pathogenic bacterial species. Studies of antibiotic resistance in clinical strains focus solely on the determination of susceptibility using disk diffusion tests and subsequent classification to the R (resistant) or S (susceptible) groups according to the guidelines of such organizations as CLSI (Clinical and Laboratory Standards Institute, 2006). Publications concerning such strains rarely explain the molecular mechanisms of resistance, i.e., the identification of specific genes by PCR amplification or hybridization. There are also no studies on the correlation between the presence of these genes and MGE, in contrast to studies on environmental *Aeromonas* strains. Hence, the comparison of the mobilome of environmental and clinical isolates of *Aeromonas* at this stage of research is virtually impossible. One can only compare the phenotypic profiles of resistance. The resistance profile of *Aeromonas* clinical and environmental strains is very similar, but additional resistance to chloramphenicol and florfenicol can be found in the latter (Michel et al., 2003). However, resistant profiles differ depending on species and geographical localization. The main reason for this is the serious lack of sufficient data about the contribution of antibiotic resistant clinical strains of *Aeromonas* that are not under epidemiological surveillance in most parts of the world. At this point it is worthwhile to refer to the term “clinical strain” itself, and answer the question what the difference between “clinical” and “environmental” strain is. While in the case of environmental strains an explanation arises spontaneously, it is no longer so obvious for clinical strains. Based on an analysis of the literature data, it can be said that the site of isolation is the essence of the definition of a clinical strain and the ability to cause disease in humans. Thus, clinical strains may be the same as environmental ones, while the opposite is not always true. The aquatic environment seems to be a “hot spot” for the transmission of antibiotic resistance caused by the selective pressure associated with excessive use of antimicrobial compounds. A wide range of ARG have been found in *Aeromonas* spp., as described in the preceding chapters. Although numerous ARG have been found on plasmids and other MGE, sulfonamide (*sul*), tetracycline (*tet*), quinolone (*qnr*), and β-lactam (*bla*) resistance genes are most common. Nevertheless, there is no correlation between one definite group of plasmids and any particular ARG. In addition, among heavy metal resistance genes, only mercury resistance genes (*mer*) have been found on MGE. They were identified among several R-plasmids that belong to IncA/C. It is worth noticing that the co-localization of heavy metal resistance and ARG on the same MGE can promote a co-selection mechanism (Pérez-Valdespino et al., 2014; Yi et al., 2014b).

It should also be noted that other MGE, such as IS, transposons or mobile integrons form a complex mobilome and may play a significant role in the dissemination of ARG. This can be explained by natural transformation, which is a general property of *Aeromonas* spp. in the environment (Huddleston et al., 2013). Frequent transformation of exogenous DNA may indicate different genetic structures of *Aeromonas* populations, including the participation of various MGE. This is consistent with previous observations in that there is no clear, detectable association between *Aeromonas* species, virulence pattern, source or origin (Tanaka et al., 2013; Martino et al., 2014). However, a review of the literature data shows a clear association between mobilome and ARG. This makes the genus *Aeromonas* a complex one and highlights the fact that there are many mechanisms of antibiotic resistance dissemination among prokaryotes. The situation is different for virulence factors. As knowledge about virulence factors and infection is incomplete, there is no clear evidence of their association with MGE. However, it has been demonstrated that the genomic plasticity of *A. salmonicida* is dependent on various IS. The majority of clinical strains, especially of *A. caviae* are considered pathogenic to humans, but they did not present all of the main known virulence factors (Janda and Kokka, 1991; Khajanchi et al., 2010; Ottaviani et al., 2011). The low prevalence of these factors suggests that pathogenicity may not depend on these virulence markers, but primarily on adaptation toward specific habitats. It should be noted that these processes also play a significant role in the distribution of strains in the environment. Originally, *Aeromonas* spp. were described as fish pathogens. Currently, these bacteria are considered emerging human pathogens, but their effective role in virulence toward humans remains controversial. This does not change the fact that the mobilome of *Aeromonas* has a considerable potential, particularly in terms of antibiotic resistance, the possibility of horizontal transfer of resistance genes, and the threat it may pose to humans.

### Conflict of interest statement

The authors declare that the research was conducted in the absence of any commercial or financial relationships that could be construed as a potential conflict of interest.
